# Mite load predicts the quality of sexual color and locomotor performance in a sexually dichromatic lizard

**DOI:** 10.1002/ece3.5689

**Published:** 2019-10-02

**Authors:** Richard W. Orton, Chase T. Kinsey, Lance D. McBrayer

**Affiliations:** ^1^ Department of Biology University of Texas at Arlington Arlington TX USA; ^2^ Department of Biology Clemson University Clemson SC USA; ^3^ Department of Biology Georgia Southern University Statesboro GA USA

**Keywords:** honest signals, parasite‐mediated selection, selection for handicap, sexual dichromatism

## Abstract

Since Darwin, the maintenance of bright sexual colors has recurrently been linked to mate preference. However, the mechanisms underpinning such preferences for bright colors would not be resolved for another century. Likely, the idea of selection for colors that could decrease the chances of survival (e.g., flashy colors that can inadvertently attract predators) was perceived as counterintuitive. It is now widely accepted that these extreme colors often communicate to mates the ability to survive despite a “handicap” and act as honest signals of individual quality when they are correlated with the quality of other traits that are directly linked to individual fitness. Sexual colors in males are frequently perceived as indicators of infection resistance, in particular. Still, there remains considerable discord among studies attempting to parse the relationships between the variables associating sexual color and infection resistance, such as habitat type and body size. This discord may arise from complex interactions between these variables. Here, we ask if sexual color in male Florida scrub lizards (*Sceloporus woodi*) is an honest signal of resistance to chigger mite infection. To this end, we use linear modeling to explore relationships between mite load, different components of sexual color, ecological performance, body size, and habitat type. Our data show that that the brightness of sexual color in scrub lizards is negatively associated with the interaction between mite load and body size, and scrub lizards suffer decreased endurance capacity with increases in mite load. Our data also indicate that mite load, performance, and sexual color in male scrub lizards can vary between habitat types. Collectively, these results suggest that sexual color in scrub lizards is an honest indicator of individual quality and further underscore the importance of considering multiple factors when testing hypotheses related to the maintenance of sexual color.

## Graphical abstract

The data submitted here help to disentangle the convoluted links between color, parasitism, and ecological performance. Collectively, the results suggest that sexual colors in male scrub lizards may be maintained by parasite‐mediated selection.

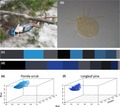

## 1 INTRODUCTION

Although initial observations of bright colors linked to sexual selection (e.g., peacock feathers) fomented much intrigue and perplexity among pioneering evolutionary biologists, it is widely held today that such colors are generally selected for because they reflect the ability to survive despite accrued disadvantages (selection for handicap; Zahavi, 1975). By extension, sexual colors are considered honest signals when they are positively correlated with the quality of other traits, such as the ability to resist disease (Hamilton & Zuk, 1982; Kodric‐Brown & Brown, 1984; Számadó, 2011; Zahavi, 1975). Honest signals are particularly important in polygynous breeding systems where males compete for access to females (Irschick, Meyers, Husak, & Le Galliard, 2008) and are often implicated in conferring male resistance to disease. Likely, the latter is due to the high heritability of genes linked to disease resistance (Bishop & Morris, 2007; Brown, Siuon, & Shero, 1995; Haldane, 1949; Mackinnon, Mwangi, Snow, Marsh, & Williams, 2005; Moritz, McCallum, Donnellan, & Roberts, 1991). However, resolving the relationships between the specific factors associated with the maintenance and drive of sexual color evolution (e.g., environmental and physiological variation) remains a difficult task and accord among results is far from ubiquitous. Even with regard to signaling of infection resistance, there are multiple standing hypotheses.

Parasitic infection is expected to result in increased constraints of signal production for low‐quality individuals because the ability to produce costly ornamentation and color is limited by an individual's ability to resist or cope with infection (Johnstone, 1995; Smith, 1991; Zahavi, 1975). As such, there have been multiple explanations as to how male quality is communicated to females during episodes of parasitic infection. For example, the “good genes” hypothesis (Hamilton & Zuk, 1982) suggests that females prefer males with elaborate sexual colors because males with the most epigamic characters have increased exposure to a wider array of parasites, thus reflecting a selection for handicap. Infection resistance is thought to be genetically determined in this hypothesis, and in turn, females that mate with males expressing elaborate sexual colors gain a selective advantage in that their offspring should confer increased resistance to infection. Similarly, Folstad and Karter (1992) provided statistical evidence for endocrinologic trade‐offs that occur between immune function and sexual color expression during infection, whereby higher levels of testosterone lead to increased expression of sexual colors but decreased infection resistance. Therefore, selection for an immunocompetence handicap allows females to immediately assess individual male quality. However, these two prevailing hypotheses are not mutually exclusive of one another and it may be important to consider environmental and physiological factors that underlie parasitic infection and sexual color expression when testing any hypothesis related to the signaling of infection resistance.

Environmental and physiological variation (e.g., microhabitat use and body size) may shape the conditions under which sexual color is maintained (Cole & Endler, 2015; Endler, 2000). For example, parasitic loads regularly differ between habitats, where several factors, including environmental parasite abundance and variation in microhabitat or substrate use, influence parasite loads (Kerr & Bull, 2006; Leu, Kappeler, & Bull, 2010; Pollock, Vredevoe, & Taylor, 2012). Perhaps more importantly, there may be interaction effects between factors related to sexual color and parasitic infection. For instance, recent evidence suggests that sexual color in male Eastern fence lizards (*Sceloporus undulatus*) is strongly associated with body size (Goodlett & Stephenson, 2019). However, larger lizards also commonly accumulate increased parasite loads, which can lead to decreased quality of sexual color (Christian & Bedford, 1995). Thus, differential exposure to parasites between habitat types and body size are likely important to include in any assessment of sexual color evolution. Although a broad consensus of the fine‐scale evolution of sexual color is unlikely because the mechanisms of sexual selection may vary across taxa, it may be informative to leverage the independent relationships of parasitic infection, body size, and sexual color across different habitat types to determine taxa‐specific constraints on sexual color. Such a study may then, in turn, be used as a framework for similar studies in additional taxa.

Sexually dichromatic lizards present ideal systems in which to explore the connections between parasitism and sexual color because of their ease of manipulation in the field and laboratory. Relationships between parasitism and performance have been extensively studied across Sceloporine lizards, in particular, which often exhibit sexual dichromatism (Garland, 1999; Huey & Dunham, 1987; Sinervo & Losos, 1991; Vitt & Pianka, 2014) that is maintained by mate preference in multiple species (Bastiaans et al., 2014; Calisi & Hews, 2007; Vinegar, 1972). Importantly, the influence of parasitic infection on individual quality can manifest as decreased performance ability (Clayton, 1991). Empirical data also show that sexual color in Eastern fence lizards is strongly associated with circulating levels of testosterone (Cox, Skelly, Leo, & John‐Alder, 2005) and thus could be compromised by parasitic infection if there are indeed endocrinologic trade‐offs between sexual color and parasitic infection as proposed by Folstad and Karter (1992).

**Figure 1:**
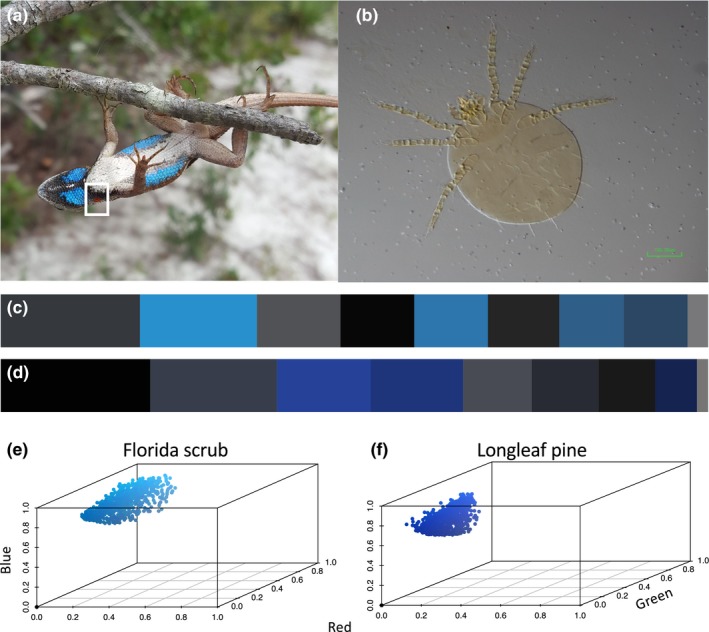
(a) Photograph of ventral side of male Florida scrub lizard taken in the field. Note the accumulation of chigger mites in the gular fold (outlined by white square). (b) Magnified image of larval *Eutrombicula cinnabaris* (chigger mite) sampled from a male scub lizard and slide‐mounted in Hoyer's medium. (c–d) Histograms of the blue color badge and black border of male scrub lizards with median brightness values collected from Florida scrub (FSC) (c) and longleaf pine (LLP) (d). In each histogram, colors have been binned into 10 values and the length of each color segment represents the proportion of each color. (e–f) Chromaticity diagrams of the blue badge color sampled for lizards with median brightness values from FSC and LLP, respectively. Note that chromaticity diagrams are shown as red‐green‐blue (RGB) values with percent blue on the *Y*‐axis, percent red on the *X*‐axis, and percent green on the *Z*‐axis

A close relative of the Eastern fence lizard, the Florida scrub lizard (*Sceloporus woodi*), is a sexually dichromatic species with sexual colors (blue color badges and black borders) that are only expressed in males (Figure 1a). Scrub lizard populations within the Ocala National Forest (ONF) of central Florida occupy both longleaf pine (LLP) and Florida scrub habitat (FSC) where they exhibit variation in substrate use between habitat types (Kaunert & McBrayer, 2015). Scrub lizards sampled in both habitat types are also commonly observed to harbor larval *Eutrombicula cinnabaris*, a terrestrial species of chigger mite (Figure 1b; Johnston & Crossley, 1996). Although ectoparasites have been shown to impose important fitness costs on hosts, including reduced performance ability (Goodman & Johnson, 2011) and diminished sexual trait expression (as reviewed by Proctor & Owens, 2000), little is known about the physiological impacts that chigger mites can impose on their hosts. Thus, scrub lizards in the ONF provide an ideal system in which to explore relationships between variables associated with sexual color and parasitic infection.

Here, we determine if sexual color in male Florida scrub lizards is an honest signal of infection resistance by simultaneously testing the “good genes” hypothesis and the hypothesis for immunocompetence handicap. We also explore the possibility that sexual color in male scrub lizards may function in signaling to competitors, rather than mates, by testing relationships between sexual color and ecological performance. Because crypsis and escape behavior are tightly linked in scrub lizards (Orton & McBrayer, 2019), suggesting that ecological performance is important for survival, we use both sprint speed and endurance as performance measures here. Researchers routinely use these two measures as indices of ecological performance in lizards given their repeatability in the laboratory (Van Berkum, Huey, Tsuji, & Garland, 1989) and their relationship to survival and reproductive success (Jayne & Bennett, 1990; Le Galliard, Clobert, & Ferrière, 2004; Peterson & Husak, 2006). Endurance, in particular, is regularly correlated with the ability to fight in lizards (Olsson, 1994). We also include two components of sexual color (badge color brightness and black border brightness) because different components of sexual color may be differentially associated with signaling during antagonistic interactions (Swierk, Ridgway, & Langkilde, 2012). For example, blue badge coloration could advertise to mates the tenure of heritable traits while the black borders of such badges could signal to competitors during antagonistic interactions. Furthermore, we investigate the contributions of habitat type and body mass toward the expression of sexual color. To this end, we use generalized linear mixed models (GLMMs) to test relationships between badge color brightness, black border brightness, mite load, endurance, sprint speed, mass, and habitat type.

## 2 MATERIALS AND METHODS

### 2.1 Fieldwork and housing

We collected 43 lizards from five LLP (*n* = 21) and six FSC stands (*n* = 22) within the ONF between 11 July 2016 and 22 August 2016. The substrate (terrestrial or vertical perch) upon which lizards were initially observed was recorded for substrate use analysis. Lizards were then captured by hand or handheld noose and chigger mite loads were counted using a standard magnifying lens. Next, lizards were transported to the laboratory at Georgia Southern University where they were immediately weighed with a digital balance and housed under standard conditions for an acclimation period of 72–86 hr prior to collecting photographs or performance data. All lizards were housed individually on sand and provided light (on a 12L:12D photoperiod from 0700 to 1900) from commercially available 75‐watt incandescent and ultraviolet lamps. This lighting system provided both light and a temperature gradient of 32–28°C to allow adequate basking and thermoregulation according to the preferred temperature range of scrub lizards (Cowles & Bogart, 1944; Neel & McBrayer, 2018). Water and food were provided ad libitum. Lizards were only disturbed immediately before taking photographs or measuring performance. Because temperature has been documented to affect coloration in *Sceloporus* (Langkilde & Boronow, 2012; Sherbrooke, de Castrucci, & Hadley, 1994), the internal body temperature of each individual was measured with a cloacal thermometer immediately before taking photographs. Measures of internal body temperature were later used to test for correlations between body temperature and color as well as between body temperature and mite load.

### 2.2 Calibrated photographs

We digitally photographed lizards in the laboratory under standardized lighting conditions in a windowless room, illuminated only by overhead fluorescent tubes. No source of auxiliary lighting was used for any photograph, and all photographs were taken from a standard distance of 0.5 meters. Lizards were photographed using a Fujifilm S20 Pro digital camera (Fujifilm) with a Fujinon super EBC 6× zoom lens. This 35‐mm camera has an effective pixel count of 6.2 megapixels and allows for manual exposure and light metering. The camera was manually adjusted for white balance and fluorescent lighting, and a standardized ISO sensitivity, shutter speed, and lens aperture were used (ISO = 200, shutter‐speed 1/25th second exposure time, and F/6.0 aperture). We photographed individuals at a resolution of 6 megapixels and a compression ratio of 1:4 (fine quality setting) before saving all images as 1,280 × 960 JPEG files. Images were then analyzed in JPEG format after linearizing pixel values. The ventral surface of each lizard was photographed against eight color‐aid basic gray scales (Color‐aid), as well as one black standard and one white standard to allow for calibration and grayscale equalization in Adobe Photoshop (as in Stevens, Párraga, Cuthill, Partridge, & Troscianko, 2007).

Measures of brightness were estimated by calculating the sum of respective reflectance percentages for red, blue, and green color channels. Thus, we here interpret brightness to be analogous to reflectance. Reflectance percentages were linearized against the slope of the reflectance curve generated from the set of standards for which reflectance values are known. For example, the white standard has a reflectance percentage of 99 and the black standard has a reflectance percentage of four. This was done for each photograph individually. This method of measuring red‐green‐blue (RGB) color was recently compared with spectrophotometric data and showed strong positive correlations between results of hue, saturation, and brightness (Orton & McBrayer, 2019). It should also be noted here that bright badge color and black borders reflect a dearth of color saturation that approaches gray, and lower brightness values reflect colors with increased hue and saturation (Orton & McBrayer, 2019). We measured brightness for the left and right color badges (under the head), the ventral–lateral stripes (coloration on the belly as seen in Figure 1a), and the black borders that outline color badges. Respective left and right measures of brightness (for badge, black border, and ventral–lateral stripes, separately) were averaged together to obtain the mean brightness for each individual for each color patch (component).

### 2.3 Performance

All performance measures were collected during the breeding season for lizards when androgen levels are thought to be increased and relatively stable in lizards (Tokarz, McMann, Seitz, & John‐Alder, 1998), and between active hours (1000 and 1400). Two trials were collected per lizard and only “successful” performance trials, defined by trials without collisions with sidewalls, pauses, or reversals, were used for analyses. The mean of two “successful” trials was used in all statistical analyses, although differences between trials typically only varied by several seconds. Lizards were warmed for one hour in an incubator at 36°C to control for confounding temperature effects. For sprint speed, each lizard was marked with nontoxic paint on the parietal scale as a landmark for digitization. Lizards were run along a custom‐built track and recorded using two Mega Speed X4^©^ high‐speed video cameras with RICOH lenses (50 mm, F/1.4 VGA) mounted on tripods (300 fps; resolution 1,080 × 1,024). We used cork substrate to reduce slippage during sprinting trials. Lizards with autotomized and broken tails were noted and excluded from any analysis. Taps on the tail and loud noises were used to coerce lizards down the track to a dark hide. To measure endurance, we ran lizards individually on a motorized pet treadmill at a belt speed of 0.20 m/s. Lizards were placed on the rubber‐cloth treadmill belt and encouraged to walk by gently tapping the lizard's tail by hand. Lizards were run until they stopped pace and failed to correct a righting response (i.e., failed to right when placed on their backs). The total run time was recorded with a commercial stopwatch and measured to the nearest 100th of a second.

### 2.4 Statistical analyses

All statistical analyses were performed in R version 3.5 and color data were visualized using R package *colordistance* (Weller & Westneat, 2019; Figure 1c–f). Two lizards were removed from the analysis of sexual color due to missing performance data. We used multiple analysis of variance (MANOVA) to determine whether variables of interest differ between LLP and FSC habitat types. Variables that did not meet the assumptions of normality and equal variance (sprint speed and mite load) were log transformed. We then constructed GLMMs for the response variables of badge color brightness, black border brightness, mite load, endurance, and sprint speed. We ran models with different response variables because analyzing color as a predictor of mite load tests the “good genes” hypothesis and analyzing mite load as a predictor of color tests a handicap for immunocompetence. For badge color brightness and black border brightness, we set opposite components (e.g., badge color or black border), mite load, mass, and interaction terms (mite load by mass and mite load by habitat type) as predictor variables (Table 1). For the model with mite load as the response, we used both color components, mass, and the interaction of mass by habitat type as predictor variables (Table 2). Because we were interested in performance measures as indices of fitness putatively indicated by variation in sexual color, we ran separate models for performance measures where we only included badge color brightness, black border brightness, and mite load as predictor variables (Table 3). In all GLMMs, stand (sampling location) was included as a random factor, and sampling date was included in models of color and mite load because mite loads can seasonally vary (Encarnação, Baulechner, & Becker, 2012). In order to assess different model functions, we used R package AICcmodavg (Mazerolle, 2019) to compute and rank AICc and QAICc for models with different link functions. Generalized linear mixed modeling was then implemented using R packages *nlme* (Pinheiro, Bates, DebRoy, & Sarkar, 2019) and *MASS*, and we used penalized quasilikelihood and log‐link functions.

**Table 1:** Results from generalized linear mixed modeling with black border brightness and badge color brightness set as response variables

Predictor variable	Black border brightness				Badge color brightness			
	*SE*	*df*	*t*‐value	*p* Value	*SE*	*df*	*t*‐value	*p* Value
Badge color brightness	0.01	26	1.79	.085	—	—	—	—
Black border brightness	—	—	—	—	0.01	26	2.16	**.039**
Mite load	0.07	26	2.44	**.022**	0.04	26	2.78	**.035**
Mass	0.24	26	1.79	.083	0.13	26	1.16	.317
Mite load × mass	0.02	26	2.13	**.043**	0.01	26	2.64	**.014**
Mite load × habitat	0.02	26	0.61	.521	0.028	26	2.17	**.039**
Sampling date	0.09	26	0.51	.559	0.081	26	0.13	.891

Note

Significant values are in bold.

**Table 2:** Results from generalized linear mixed modeling with endurance and sprint speed set as response variables

Predictor variable	Mite load			
	*SE*	*df*	*t*‐value	*p* Value
Badge color brightness	0.01	26	0.68	.501
Black border brightness	0.01	26	0.43	.665
Mass	0.19	26	0.51	.616
Mass × habitat	0.06	26	3.94	**.001**
Sampling date	0.17	26	0.89	.382

Note

Significant values are in bold.

**Table 3:** Results from generalized linear mixed modeling with mite load set as the response variable

Predictor variable	Endurance				Sprint speed			
	*SE*	*df*	*t*‐value	*p* Value	*SE*	*df*	*t*‐value	*p* Value
Badge color brightness	0.01	28	0.46	.651	0.01	28	1.78	.081
Black border brightness	0.01	28	0.77	.448	0.01	28	0.09	.931
Mite load	0.01	28	2.34	**.026**	0.01	28	1.49	.147

Note

Significant values are in bold.

To test for significant associations between body temperature and sexual color components, as well as mite load and body temperature, we used separate Pearson's correlations. These correlations were done separate of linear models to increase sample size because multiple individuals were dropped from linear models due to missing performance data. We also used a Pearson's correlation to test the relationship between badge color brightness and the brightness of the ventral–lateral stripes because preliminary analyses indicated that these color components are similar and thus likely share similar functional significance. Last, we used a Fisher's exact test to determine the contingency of substrate use (vertical vs. terrestrial) on habitat type.

## 3 RESULTS

**Figure 2:**
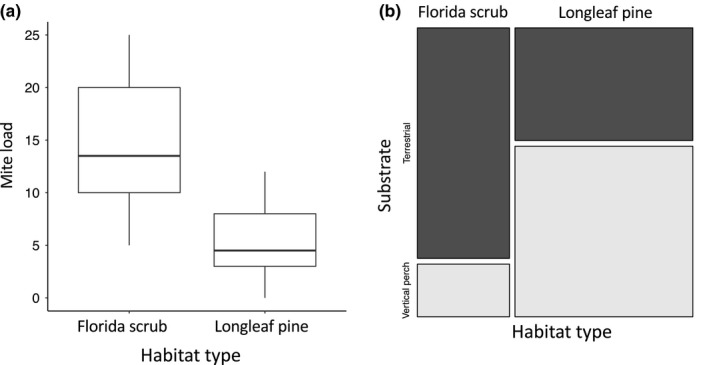
(a) Box plot showing mite load (range, quantile, and median) for longleaf pine (LLP) and Florida scrub (FSC) habitat types. Median mite load harbored by male scrub lizards is higher in FSC (*F*_(1,38)_ = 31.76, *p* = .001). (b) Mosaic plot showing the proportion of substrate types for which male scrub lizards were initially encountered for LLP and FSC habitat types. The substrate type upon which scrub lizards were encountered is contingent on habitat type (odds ratio = 6.52, *p* = .001)

**Figure 3:**
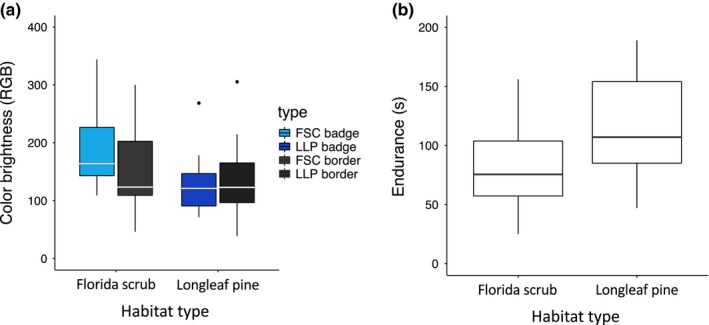
(a) Grouped box plot showing male Florida scrub lizard badge color brightness and black border brightness (range, quantile, and median) for longleaf pine (LLP) and Florida scrub (FSC) habitat types. Badge color and black border groups are colored according to the legend. Scrub lizard badge color is darker in LLP (*F*_(1,38)_ = 12.32, *p* = .001) although black border brightness of scrub lizards does not significantly vary between habitat types (*F*_(1,38)_ = 0.17, *p* = .675. (b) Box plot showing scrub lizard endurance (range, quantile, and median) for LLP and FSC habitat types. Scrub lizards collected from LLP have greater endurance (*F*_(1,38)_ = 6.57, *p* = .014), although sprint speed does not significantly vary between habitat types (*F*_(1,38)_ = 0.155, *p* = .659)

Results from MANOVA show that mite load (*F*_(1,38)_ = 31.76, *p* = .001; Figure 2a) is significantly higher in FSC habitat, compared with LLP habitat, while results from the Fisher's exact test show that the substrate upon which lizards were encountered in the field was contingent on habitat type (odds ratio = 6.52, *p* = .001; Figure 2b). Additional MANOVA results show that badge color brightness (*F*_(1,38)_ = 12.32, *p* = .001; Figure 3a) and endurance (*F*_(1,38)_ = 6.57, *p* = .014; Figure 3b) also significantly vary between FSC and LLP habitats, although black border brightness (*F*_(1,38)_ = 0.17, *p* = .675), sprint speed (*F*_(1,38)_ = 0.155, *p* = .659), and mass (*F*_(1,38)_ = 1.364, *p* = .178) do not. Before assessing the relationships between color and other variables, we tested the relationship between badge color brightness and the brightness of the ventral–lateral stripes and found that the different color patches are highly and significantly correlated (Pearson's coefficient = 0.509, *p* = .001). Thus, we only focus this study on badge color brightness and the black border of the badges because these results suggest that ventral‐lateral stripes and badges are likely to bear similar relationships with mite load and have similar biological functions.

**Figure 4:**
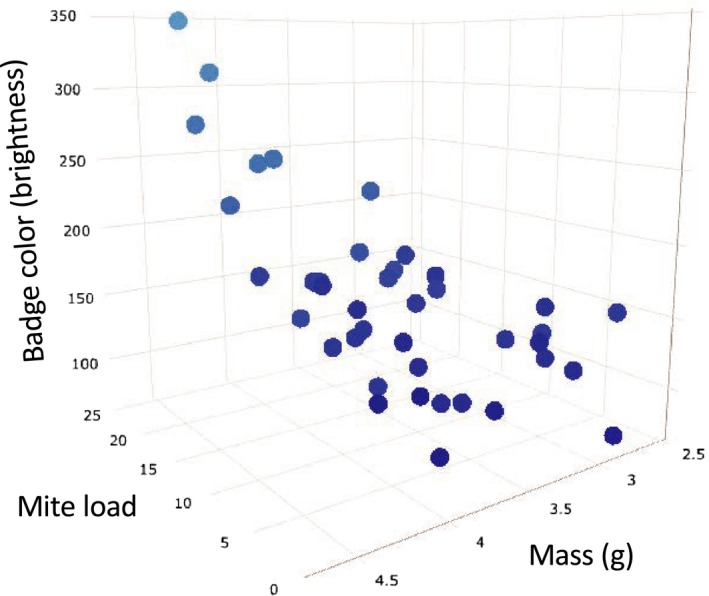
Three‐dimensional plot showing the relationships between mass (*X*‐axis), mite load (*Y*‐axis), and badge color brightness (*Z*‐axis) in male Florida scrub lizards. Individual points are denoted in the predicted badge color and are predicted by mass and mite load, where the brightness of scrub lizard badge color increases as mass and mite load increase (*p* = .014, *R* ^2^ = .25). In particular, smaller scrub lizards with fewer mites tend to have the darkest colors and larger lizards with more mites have brighter colors that approach gray

**Figure 5:**
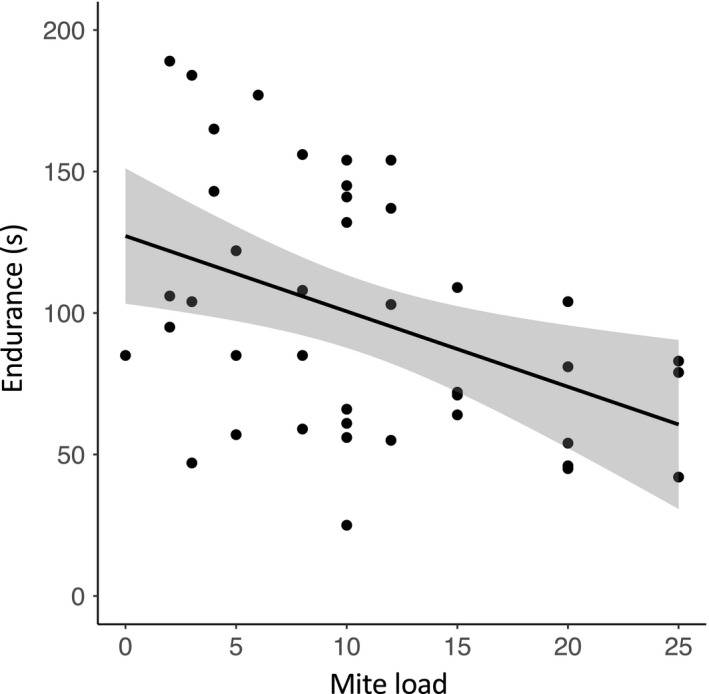
Scatter plot showing the predicted negative relationship between mite load and scrub lizard endurance (*p* = .026, *R* ^2^ = .24). Scrub lizards with increased mite loads have less endurance than scrub lizards with fewer mites. The gray cloud surrounding the line of best fit is (±) 1 *SE*

**Figure 6:**
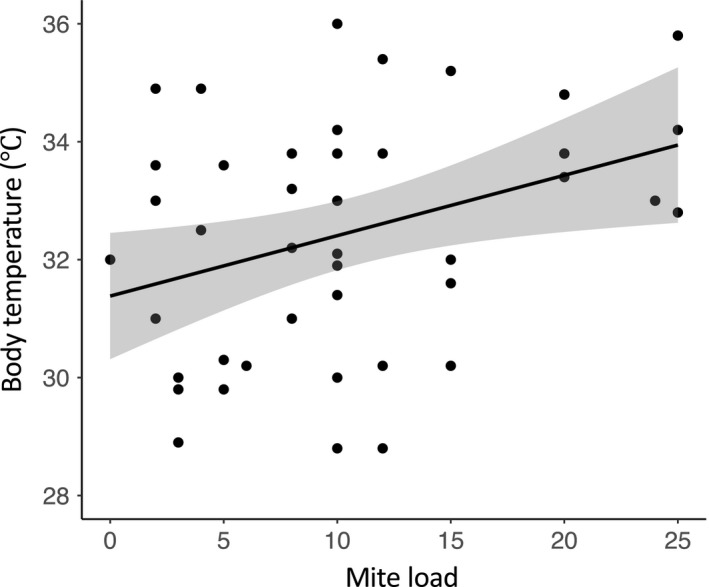
Scatter plot showing the positive relationship between mite load and scrub lizard internal body temperature (Pearson's coefficient = 0.36, *p* = .017). In captivity, scrub lizards with increased mite loads tend to have higher internal body temperatures than scrub lizards with fewer mites. The gray cloud surrounding the line of best fit is (±) 1 *SE*

Results from GLMMs (Tables 1, 2, 3) indicate that mite load is associated with badge color brightness (*p* = .035, *R* ^2^ = .22) and black border brightness (*p* = .022, *R* ^2^ = .18). However, although black border brightness predicts badge color brightness (*p* = .039, *R* ^2^ = .15), badge color brightness is not a predictor of black border brightness (*p* = .085, *R* ^2^ = .06). Notably, mass alone predicts neither badge color brightness nor black border brightness, although significant interaction effects between mass and mite load indicate that mite load has a larger effect on badge color brightness (*p* = .014, *R* ^2^ = .25; Figure 4) and black border brightness (*p* = .043, *R* ^2^ = .19) of larger lizards. Furthermore, the significant interaction term between mite load and habitat type indicates that relationships between mite load and badge color brightness vary between FSC and LLP (*p* = .039, *R* ^2^ = .26). Conversely, the only significant predictor of mite load is the interaction between mass and habitat type (*p* = .001, *R* ^2^ = .46; Table 2). Furthermore, sampling date is not a predictor of badge color brightness (*p* = .891, *R* ^2^ = .001), black border brightness (*p* = 0.559, *R* ^2^ = .014), or mite load (*p* = .382, *R* ^2^ = .033). Generalized linear mixed modeling also indicates that mite load is a predictor of endurance (*p* = .026, *R* ^2^ = .24; Figure 5), but not sprint speed (*p* = .147, *R* ^2^ = 0.11). However, neither badge color brightness nor black border brightness is a predictor of endurance capacity (*p* = .651, *R* ^2^ = 0.01 and *p* = .448, *R* ^2^ = .03 respectively) or sprint speed (*p* = .08, *R* ^2^ = .13 and *p* = .931, *R* ^2^ = .01 respectively; Table 3). Last, we found that body temperature is associated with mite load (Pearson's coefficient = 0.36, *df* = 42, *p* = .017) (Figure 6). Body temperature is not, however, significantly correlated with badge color brightness (Pearson's coefficient = 0.27, *df* = 42, *p* = .077) or black border brightness (Pearson's coefficient = 0.16, *df* = 42, *p* = .311).

## 4 DISCUSSION

The presence of blue coloration on the ventral surface of male, but not female scrub lizards, suggests that dichromatism is important to maintain despite the risk of inadvertently cuing visual predators. This dichromatism is further observed in other lizards and species of *Sceloporus*, where it has been determined to be an honest signal of individual quality (Calisi & Hews, 2007; Cooper & Burns, 1987). Notably, sexual color in male scrub lizards is not a predictor of mite load, as would be expected given the “good genes” hypothesis (Hamilton & Zuk, 1982), nor is sexual color a predictor of either measure of ecological performance measured here (sprint speed and endurance). If the function of sexual color in male scrub lizards is to communicate the ability to fight and defend territory to other males, it might be expected that sexual color would be correlated with endurance or sprint speed. However, mite load is a predictor of sexual color in male scrub lizards. Together, these results suggest that sexual color in male scrub lizards is an honest signal that potentially communicates individual quality to females through a handicap for immunocompetence (Folstad & Karter, 1992).

Expounding upon Zahavi's (1975) hypothesis for a selection for handicap, as well as Hamilton and Zuks (1982) “good genes” hypothesis, Folstad and Karter (1992) proposed the presence of a negative feedback loop between parasite burden and sexual color expression, where color expression is testosterone‐dependent and plastic. More recent evidence also shows that testosterone can inhibit immune response by redirecting resources toward sexual trait expression (Buchanan, Evans, & Goldsmith, 2003; Capaul & Dieter, 2003; Evans, Goldsmith, & Norris, 2000; Faivre, Grégoire, Préault, Cézilly, & Sorci, 2003; Gasparini et al., 2009; Poiani, Goldsmith, & Evans, 2000; Verhulst, Dieleman, & Parmentier, 1999; Wedekind & Folstad, 1994). Thus, it is interesting to note that expression of blue badge coloration and black badge borders in a close relative of the scrub lizard, the Eastern fence lizard, is mediated by circulating levels of testosterone (Cox et al., 2005), because the evolution of sexual color by sexual selection requires that the costs of sexual color production must be linked to regulatory elements that share pathways with other fundamental physiological processes (Hill, 2011).

In male scrub lizards, black border brightness is a predictor of badge color brightness, a predominately blue color patch, although badge color brightness does not predict black border brightness. The expression and brightness of blue coloration in animals, which is often linked to sexual selection in lizards (as reviewed by Umbers, 2013), results from the reflective properties of iridophore cells that are underlain by melanophores (Kottler et al., 2014; Kuriyama, Miyaji, Sugimoto, & Hasegawa, 2006). The production of melanin not only underpins dark coloration across many taxa (reptiles; Cox et al., 2005; Cox, Zilberman, & John‐Alder, 2008; Rosenblum, Hoekstra, & Nachman, 2004, birds; Hill & Brawner, 1998; Lindsay, Webster, & Schwabl, 2011, fishes; Kittilsen et al., 2009), it also requires the influence of testosterone (as reviewed by Jawor & Breitwisch, 2003) and 5α‐dihydrotestosterone (Pollock, Feigin, Drazenovic, & John‐Alder, 2017), and plays a key role in immune response (Andersen et al., 2005; Catania et al., 1996; Taherzadeh et al., 1999). As such, sexual color in scrub lizards may be mediated by the expression of melanin, which is linked to the regulation of testosterone.

By leveraging linear models with different combinations of predictor and response variables, we were able to determine here that sexual color in male scrub lizards is a response and not a predictor of mite load. If the inverse were indicated, where elaborate color was a predictor of increased mite load, then sexual color in scrub lizards would support Hamilton and Zuk's (1982) hypothesis, which proposes that individuals with the most elaborate secondary sexual characteristics are more prone to exposure to parasites. Rather, we infer from our data, in tandem with empirical data measured in Eastern fence lizards and scrub lizards (Cox & John‐alder, 2007; Cox et al., 2005, 2008; Gowan, McBrayer, & Rostal, 2010), that links between sexual color and mite load in male scrub lizards is putatively mediated by testosterone regulation, as in Folstad and Karter's handicap for immunocompetence (1992). In turn, female scrub lizards may be able to directly asses individual male quality. However, there may be additional factors to consider when exploring the maintenance of sexual color in scrub lizards.

Although many studies have investigated associations between parasitism, sexual color, and ecological performance, accord among results is not ubiquitous. One explanation regarding this lack of consensus may be the variation in environmental parasite abundances observed between different habitat types or microhabitats (Biaggini, Berti, & Corti, 2009; Johnston & Crossley, 1996). Scrub lizard mite loads, for example, are markedly higher in FSC habitat compared with LLP habitat in the ONF (Figure 2a). Although this variation could be due to differences in scrub lizard population density, it is more likely related to differences in substrate use driven by management practice. In the ONF, vertical perches (pine trees and turkey oaks) are removed from FSC, but not from LLP (McCoy, Styga, Rizkalla, & Mushinsky, 2012). Because scrub lizards in LLP are most often encountered on vertical perches despite the availability of terrestrial substrates (Kaunert & McBrayer, 2015), it is feasible that the removal of perches influences variation in scrub lizard substrate use between habitat types (Figure 2b). Larval chigger mites are also primarily terrestrial (Johnston & Crossley, 1996), and thus, predominantly terrestrial scrub lizards are likely to accumulate increased mite loads compared with lizards that spend a larger proportion of time on vertical perches. Variation in mite load between habitats may then differentially impact variables associated with sexual color.

In addition to increased mite loads, male scrub lizards in LLP also have darker sexual colors (Figure 3a) and increased endurance (Figure 3b) when compared to lizards in FSC. This may deem it worthy to explore interactions between habitat type and other variables previously acknowledged to influence sexual color when testing relevant hypotheses. Linear modeling indicates that the interaction between habitat type and mite load predicts male badge color brightness in scrub lizards—the relationships between mite load and badge color brightness vary between LLP and FSC. This could suggest the masked influence of additional variables that vary between habitat types such as thermal quality of habitat or scrub lizard body condition. By extension, it may also be important to test interactions between mite load and other variables known to influence sexual color. For instance, older and larger lizards of the genus *Sceloporus* have been shown to exhibit increased inflammatory responses to *Eutrombicula* infection (Goldberg & Holshuh, 1992) and thus may be a principal interaction to account for regarding sexual color evolution.

Studies exploring sexual color evolution in lizards often determine that older and larger lizards have decreased sexual color expression (Christian & Bedford, 1995; Schall & Marghoob, 1995). Because melanin production is also negatively correlated with age (Soulsbury et al., 2018), and mass and age are positively correlated in lizards (Shine & Charnov, 1992), it might then be expected that as mass in scrub lizards increases, melanin production decreases, leading to increases in the brightness of sexual colors (i.e., decreased color expression). However, results presented herein show that mass, as a stand‐alone predictor variable, does not predict the brightness of badge color or black borders of male scrub lizards. Rather, badge color brightness (Figure 4) and black border brightness in scrub lizards are predicted by the interaction between mass and mite load—larger lizards with more mites have the brightest (i.e., tending toward gray) sexual colors. This interaction likely echoes the increased inflammatory responses observed in older individuals within *Sceloporus* (Goldberg & Holshuh, 1992) and highlights the importance of considering interactions between variables when endeavoring to resolve the drive and maintenance of sexual color. Although evolutionary change in morphological and performance variables tends to scale positively with the evolution of body size in lizards (Losos, 1990), variation in parasitic infection between habitat types and individuals may lead to unanticipated outcomes. Such impacts may further vary according to which correlated traits are assessed.

It may be somewhat peculiar that mite load is a predictor of endurance (Figure 5), but not sprint speed. Potentially, this is because the design features needed to maximize different performance traits can be difficult to reconcile within a single phenotype (Losos, 1990; Vanhooydonck, Damme, & Aerts, 2001). The different muscle types required to meet various optima typically acquire energy from different sources; slow oxidative muscles rely on blood oxygen levels while fast glycolytic muscles, which are involved in anaerobic performance such as sprinting, rely on muscle glycogen reserves. One expected impact of ectoparasitic infection by chigger mites is exsanguination, which can lead to lower hematocrit levels and anemia (Pryor & Casto, 2015). Hence, ectoparasitic infection might disproportionately affect muscles that depend on blood oxygen levels, having greater consequences on endurance than on spiriting. Although we are currently unable to resolve this specific discrepancy here, the observed negative impacts of mite load on endurance capacity may further establish that chigger mites can have negative impacts on individual quality. Furthermore, neither badge color brightness nor black border brightness is a predictor of either measure of ecological performance. This may suggest that sexual color in male scrub lizards does not function to signal to conspecifics the ability to fight. However, it is also possible that in scrub lizards, other ecological performance measures, such as bite force, are more closely intertwined with the outcomes of antagonistic interactions. We acknowledge that this would be an interesting avenue to explore in future studies, as would testing relationships between performance and other color metrics such as patch area.

In ectotherms, expression of structural colors may require adequate body temperatures (Bajer, Molnar, Török, & Herczeg, 2012; Hettyey, Crochet, Merilä, Herczeg, & Laurila, 2009; Langkilde & Boronow, 2012). Recent studies report that higher body temperatures are related to more reflection in the UV and blue range (Bajer et al., 2012). In a particular study assessing sexual color in Eastern Fence lizards, male sexual color changed from green to blue when the lizards were exposed to increased temperatures (Langkilde & Boronow, 2012). We did not determine that body temperature was related to badge color brightness or black border brightness in the present study. Likely, this is because the temperature gradients provided in this study encompassed scrub lizard operative temperatures, within which they were permitted to freely thermoregulate (Neel & McBrayer, 2018). However, lizards with increased mite loads did have higher body temperatures in the laboratory (Figure 6). We anticipate that infected lizards may be behaviorally staging immune responses, which may further indicate the impact of chigger mite infection on scrub lizards. Because there is little to no evidence that chigger mites induce physiological effects on reptilian hosts, this may be a minor, yet important result of the study presented herein.

Collectively, our results reveal that sexual color (badge color black border brightness) in male scrub lizards is a response to mite load, suggesting that the immunosuppressive effects of testosterone may facilitate trade‐offs between immune response and melanin production in male scrub lizards, as has been determined among a range of taxa including Sceloporine lizards (Cox & John‐alder, 2007; Folstad & Karter, 1992). Inasmuch as, female scrub lizards (or male competitors) may be able to immediately assess male individual color according to brightness of sexual color. Endurance capacity is also predicted by mite load, further implicating the negative impacts of chigger mites on scrub lizard individual quality. The data and interactions described here complement a handful of studies showing significant correlations between mite load, endurance capacity, and badge color (Main & Bull, 2000; Weiss, 2006) and emphasize the importance of simultaneously considering the impact of body size and habitat type when testing hypothesis regarding sexual color evolution and parasitic infections.

## CONFLICT OF INTEREST

None declared.

## AUTHOR CONTRIBUTIONS

RWO, CTK, and LDM conceived, designed, and performed this experiment. Additionally, RWO, CTK, and LDM analyzed the data used for statistical analyses and wrote the manuscript.

## Data Availability

Data and associated R scripts have been uploaded to Dryad as of 9 September 2019. Data from: Mite load predicts the Quality of Sexual Color and Locomotor Performance in a Sexually Dichromatic Lizard. Data Files: honest_signals (CSV file), sex_color (R script). DOI: https://doi.org/10.5061/dryad.2n3fd2p.
